# Gradient boosting with knockoff filters: a biostatistical approach to variable selection

**DOI:** 10.1186/s12859-025-06215-z

**Published:** 2025-11-25

**Authors:** Amr Mohamed, Kevin H. Lee

**Affiliations:** 1 College of Health Sciences, The University of Memphis, 3720 Alumni Ave, Memphis, TN 38152 USA; 2 Department of Statistics, Western Michigan University, 1903 W Michigan Ave, Kalamazoo, MI 49008 USA

**Keywords:** Variable selection, Knockoffs, LightGBM, Gradient boosting, SHAP

## Abstract

As data complexity and volume increase rapidly, efficient statistical methods for identifying significant variables become crucial. Variable selection plays a vital role in establishing relationships between predictors and response variables. The challenge lies in achieving this goal while controlling the False Discovery Rate (FDR) and maintaining statistical power. The knockoff filter, a recent approach, generates inexpensive knockoff variables that mimic the correlation structure of the original variables, serving as negative controls for inference. In this study, we extend the use of knockoffs to Light Gradient Boosting Machine (LightGBM), a fast and accurate machine learning technique. Shapely Additive Explanations (SHAP) values are employed to interpret the black-box nature of machine learning. Through extensive experimentation, our proposed method outperforms traditional approaches, accurately identifying important variables for each class. It offers improved speed and efficiency across multiple datasets. To validate our approach, an extensive simulation study is conducted. The integration of knockoffs into LightGBM enhances performance and interpretability, contributing to the advancement of variable selection methods. Our research addresses the challenges of variable selection in the era of big data, providing a valuable tool for identifying relevant variables in statistical modeling and machine learning applications.

## Introduction

Variable selection is arguably the most challenging part of model building in regression, classification, and clustering problems. Selecting the right variables is one of the toughest challenges in modeling, especially when dealing with high-dimensional data. Too many irrelevant or redundant variables can cloud the real relationships and reduce model performance. Traditional approaches like stepwise regression and Lasso try to address this, but they often struggle with balancing false discoveries and interpretability.

The knockoff filter [1, 2] is a more recent solution that generates synthetic variables to help control the false discovery rate (FDR) while keeping statistical power high. At the same time, machine learning models like LightGBM have become widely used for their efficiency and predictive strength, but their "black box" nature makes it difficult to interpret their decisions.

In this study, we integrate knockoffs with LightGBM to improve variable selection, replacing traditional importance measures with SHAP values for better interpretability. Our simulations and real-world microarray dataset analysis show that this approach effectively identifies significant variables while keeping FDR low. This method provides a practical, scalable, and interpretable solution for working with complex datasets.

Related work has adapted the knockoff framework to boosted tree models, including the knockoff tree approach, which is conceptually aligned with the methodology considered here[6].

## Methodology

### Light gradient boosting machine (LightGBM)

Microsoft introduced LightGBM [7] in 2017 as a Gradient Boosting Decision Trees (GBDTs) algorithm that enhances the efficiency and scalability of boosted tree models like XGboost, while maintaining high performance. LightGBM is suitable for various machine learning tasks, including classification and regression. Its primary objective was to address the challenges faced by GBDT in handling big data, particularly the trade-off between accuracy and efficiency. GDBT is an ensemble method for decision trees, which are trained in sequence. In each iteration, the GBDT learns the decision trees by fitting the negative gradients.

Consider we have the data $D = {(x_i, y_i)}: i=1,\ldots , n$, where $x_i \in \mathbb {R}^{p}$ and $y_{i} \in \mathbb {R}$, that is *n* observations and *p* features, with a response variable *y*. The result given by an ensemble generalized model is:

1 $$\begin{aligned} \hat{y}_{i} = \phi (x_i) = \sum ^{K}_{k=1} f_{K} (x_i) \end{aligned}$$

$f_k$ is a single decision tree, and $f_k (x_i)$ is the score given by the $k^{th}$ tree to the $i^{th}$ observation in the training data. The objective is to minimize the following loss function $\mathcal {L}$ in order to choose functions $f_k$.

2 $$\begin{aligned} \mathcal {L} (\phi ) = \sum ^{n}_{i} l(y_i, \hat{y_i}) + \sum ^{K}_{k=1} \Omega (f_k) \end{aligned}$$

Where $\Omega (f_k)$ is the penalty term used for regularization to avoid overfitting.

3 $$\begin{aligned} \Omega (f_k) = \gamma T + \frac{1}{2} \lambda || \mathbf{{w}} ||^{2} \end{aligned}$$

Where $\gamma$ and $\lambda$ are parameters controlling the penalty for preventing overfitting, *T* is the number of leaves and $\textbf{w}$ is the $k^{th}$ tree’s leaf node weight. Then the predictive value $\hat{y}_i^{(t)}$ after the $t^{th}$ iteration is:

4 $$\begin{aligned} \hat{y}_i^{(t)} = \hat{y}_i^{(t-1)} + f_{t} (x_i) \end{aligned}$$

Therefore, the objective function at iteration *t* can be formulated as:

5 $$\begin{aligned} \mathcal {L}^{(t)} = \sum _{i} l( y_i, \hat{y}_i^{(t-1)} + f_{t} (x_i)) + \Omega (f_t) \end{aligned}$$

By the second-order Taylor expansion, the function can be simplified and approximated, then the formula for the loss reduction after the tree split at a given node is:

6 $$\begin{aligned} \mathcal {L}^{(t)} \approx \sum ^{n} \left[ l(y_i,\hat{y}_i^{(t-1)}) + g_i f_t(x_i) + \frac{1}{2} h_i f_t^2 (x_i) \right] + \Omega (f_t) \end{aligned}$$

Where $g_i$ is the first order derivative and $h_i$ is the second order derivative:

7 $$\begin{aligned} g_i = \partial _{\hat{y}_i^{(t-1)}} l (y_i, \hat{y_i}^{(t-1)}) , \qquad h_i = \partial ^{2}_{\hat{y}_i^{(t-1)}} l (y_i, \hat{y_i}^{(t-1)}) \end{aligned}$$

Because the previous $(t-1)$ trees’ residual errors have minimal effect on the modification of the objective function, Eq. 5 can be modified as follows:

8 $$\begin{aligned} \mathcal {L}^{(t)} = \sum _{i} \left[ g_i f_t(x_i) + \frac{1}{2} h_i f_t^2 (x_i) \right] + \Omega (f_t) \end{aligned}$$

After the classification process is done, all instances that belong to the same leaf node can be reassembled as:

9 $$\left\{ {\mathcal{L}} \right\}(t) \approx \sum\limits_{{j = 1}}^{T} {\left[ {\left( {\sum\limits_{{i \in I_{j} }} {g_{i} } } \right)w_{j} + \frac{1}{2}\left( {\sum\limits_{{i \in I_{j} }} {h_{i} } + \lambda } \right)w_{j}^{2} } \right]} + \gamma {\text{ }}$$

Where $I_j$ is the set of samples that $j^{th}$ leaf node. Therefore, the optimal weight when the derivative of the objective function equal to 0 is:

10 $$\begin{aligned} w^*_j = - \frac{\sum _{i \in I_j} g_i}{\sum _{i \in I_j}h_i + \lambda } \end{aligned}$$

When $w^*_j$ = $w_j$, the objective function is:

11 $$\begin{aligned} \mathcal {L} = - \frac{1}{2} \sum _{j=1}^{T} \frac{(\sum _{i \in I_j} g_i)^2}{\sum _{i \in I_j} h_i + \lambda } + \gamma K \end{aligned}$$

The information gain of the objective function at each split can be calculated by

12 $$\begin{aligned} Information \; Gain = \frac{1}{2} \left[ \frac{(\sum _{i \in I_L} g_i)^2}{\sum _{i \in I_L} h_i + \lambda } + \frac{(\sum _{i \in I_R} g_i)^2}{\sum _{i \in I_R} h_i + \lambda } - \frac{(\sum _{i \in I} g_i)^2}{\sum _{i \in I} h_i + \lambda } \right] \end{aligned}$$

Where *I* is a subset of the observations available in the current node (before the split), $I_L$ and $I_R$ are the subset of observations available in the left and right nodes (after the split). Information gain measures how much the objective function improves (or reduces) when a split is made, which is the difference between pres-split and post-split. A higher information gain indicates a more meaningful split.

Traditional GBDT methods involve scanning all data points for each feature to estimate information gain from potential splits, leading to computational complexity that grows with the number of features and instances. To mitigate this issue, LightGBM aims to reduce the number of features and instances while ensuring careful sampling. To achieve this, the algorithm introduces two innovative methods, one of which is gradient-based one-side sampling (GOSS). To enhance the efficiency and effectiveness of the GBDT algorithm, one approach is to sample data points based on their information gain. However, utilizing sample weights is not feasible in GBDT since it lacks the concept of sample weights. This challenge is addressed by GOSS, which leverages the gradient of the instances to gain deeper insights into information gain. The gradient of each data point provides valuable information regarding its importance in the subsequent boosting step. By definition, instances with large gradients are undertrained and contribute significantly to information gain, while those with small gradients are well-trained with minimal training error. Therefore, downsampling instances based on their gradients presents a superior solution. In other words, this approach involves retaining instances with gradients exceeding a predefined threshold while randomly discarding instances with smaller gradients.

In high-dimensional data, it is common to encounter sparse features, some of which are exclusive, meaning they never have nonzero values together. To address this, the LightGBM algorithm incorporates an additional technique known as Exclusive Feature Bundling (EFB) alongside the GOSS optimization for training samples. EFB allows the bundling of these exclusive features into a single feature, effectively reducing dimensionality. This approach is employed to enhance the training speed while maintaining model accuracy. By bundling exclusive features, the complexity of creating feature histograms becomes proportional to the number of feature bundles rather than the number of individual features. This optimization further contributes to the efficiency of the algorithm without compromising the accuracy of the model. The histogram algorithm is applied to discretize successive floating-point eigenvalues into *k* integers and create a histogram of width *k*. When traversing the data, the statistic is accumulated in the histogram according to the discretized value as an index. After traversing the data once, the histogram accumulates the required statistic and then traverses to find the optimal segmentation point according to the discrete value of the histogram. This way is more efficient than uniformly random sampling and can lead to a more accurate information gain estimation. The instances are sorted according to the absolute value of their gradients. Then, GOSS samples $A \%$ of the total samples with the highest gradient and $B\%$ of examples from the remaining $(1-A)\%$. Then, the gradients of $B\%$ of examples are multiplied by $(1-A)/B$ which amplifies the smaller gradients, to ensure that the original distribution does not alter much, and prevent the next boosting step from ignoring these instances with smaller gradients completely. The subset *B* is discretionarily selected with size b $\times$ $|A^c |$ [4]. This reduces the number of training examples at each boosting step and consequently reduces memory, and training time. The variance gain of splitting the instances over subsets *A* and *B* can be defined by the following equation:

13 $$\begin{aligned} \tilde{V}_j(d) = \frac{1}{n} (L_1 + L_2) \end{aligned}$$

Where variables $L_1$ and $L_2$ are defined as follows:

14 $$\begin{aligned} L_1 = \frac{\left(\sum _{x_i \in A_l} g_i + \frac{1-a}{b} \sum _{x_i \in B_l} g_i\right)^2}{n_l^j (d)} \end{aligned}$$

15 $$\begin{aligned} L_2 = \frac{\left(\sum _{x_i \in A_r} g_i + \frac{1-a}{b} \sum _{x_i \in B_r} g_i\right)^2}{n_r^j (d)} \end{aligned}$$

**Fig. 1:**
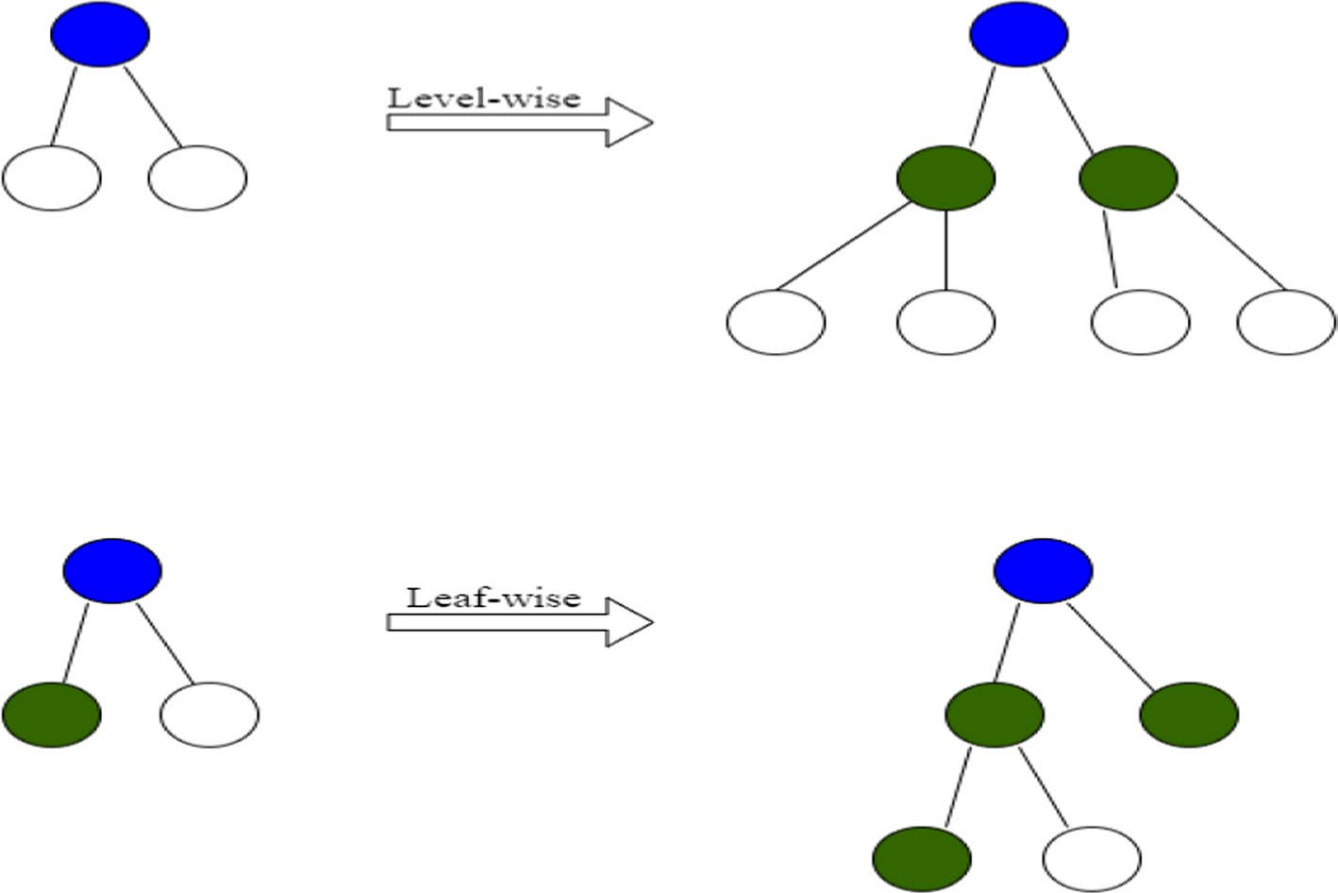
Leaf-wise vs. level-wise

Where $g_i$ is the negative gradient of the loss function with respect to the output of the GBDT model. $A_l = \{x_i \in A: x_{ij} \le d\}$, $A_r = \{x_i \in A: x_{ij}> d\}$, $B_l = \{x_i \in B: x_{ij} \le d\}$, $B_r = \{x_i \in B: x_{ij}> d\}$ and $\frac{1-a}{b}$ is a normalizing constant for the sum of gradients. LightGBM offers another advantage by employing a leaf-wise strategy for splitting trees, unlike traditional boosting algorithms that use a level-wise approach. Figure 1 illustrates the distinction between level-wise and leaf-wise splits. In level-wise splitting, the leaves at the same layer are simultaneously divided. This technique is inefficient as it treats all leaves of the same layer uniformly, resulting in overfitting due to unnecessary search and splitting of many leaves with lower split gain. On the other hand, the leaf-wise strategy employed by LightGBM is more efficient. It selects the leaf with the highest split gain from the current layer to split, ensuring optimal utilization of resources. Consequently, the leaf-wise strategy outperforms the level-wise strategy. Additionally, LightGBM controls the complexity of the tree by setting a maximum depth, preventing excessive growth and addressing the issue of overfitting. This way, the algorithm strikes a balance between capturing valuable information and avoiding unnecessary complexity in the network.

Hyperparameters play a crucial role in determining the accuracy of a model. Hence, it is essential to establish the important parameters and define their acceptable value ranges prior to utilizing the LightGBM algorithm. LightGBM encompasses several key parameters, including the number of leaves per tree, the learning rate (which controls the iteration speed), the maximum depth of each tree (to address overfitting), the minimum number of records a leaf can contain, the fraction of randomly selected features for tree construction in each iteration, and the ratio for retaining large gradient data and the retain ratio for small gradient data (specifically used in GOSS boosting). By appropriately setting these important parameters, the model accuracy can be significantly influenced and optimized. Among various global optimization algorithms, Bayesian Optimization has gained popularity due to its efficient convergence and superior performance. Bayesian Optimization has emerged as a robust technique for solving optimization problems across diverse application domains. Hence, in our work, we used Bayesian optimization [5, 10] to find optimal hyperparameters.

### Variable selection via Knockoffs

The knockoff filter was proposed in 2015 by Barber and Candès [1], it is a general framework for controlling the false discovery rate when performing variable selection. The idea is to be able to discover the truly associated predictors with the response variable. The knockoff filter operates by generating knockoff variables that are designed to mimic the correlation structure found within the original data. Creating knockoffs is cheap and their construction does not require collecting any new data. The knockoffs serve as negative controls and they allow identifying the important variables that are related to the response variable while controlling the expected fraction of the false discovery proportion - FDR. The knockoff method selects the original variables that are better than their corresponding knockoff copies, based on some measures of feature importance that can be computed with a variety of popular methods. The knockoff filter has been used and shown to ensure accurate FDR control, which cannot be achieved using the traditional methods. The method proposed in 2015 [1] works under the condition that, for a fixed design matrix $\mathbf{{X}} \in \mathbb {R}^{n \times p}$, where $n> p$ and the response variable *y* follows a linear Gaussian model. This work has been extended in 2018 [2] to model-X and high dimensional knockoffs. In this section and the next subsections, we will adapt the model-X knockoffs to the boosted decision tree model. Model-X knockoffs for a family of *p* random variables $\mathbf{{x}} = (x_1, x_2, \dots , x_p)$ are a new family of random variables $\mathbf{{k}} = (k_1, k_2, \dots , k_p)$ , where each $x_j$ is a random variable that represents a feature, and each $k_j$ is its corresponding knockoff feature. The construction of these knockoff variables requires two properties:

1. For any subset $S \subset \{1, \dots , p \}$, $(X, K)_{swap(S)} {\mathop {=}\limits ^{d}} (X, K)$. This property states that swapping the each original $x_j$ with its knockoff $k_j$ for each $j \in S$ leaves the join distribution invariant.
2. $K \perp \!\!\!\perp Y\; |\; X$, this conditional independence is guaranteed if *K* is constructed without looking at the response *Y*.

The knockoff filter method has three main components to accurately control the FDR.

- *Constructing knockoff* The knockoff variables are constructed to mimic the correlation structure of the original features. First, the Gram matrix $\mathbf{{\Sigma = X^{T} X}}$ of the original features is computed, after normalizing each feature such that $\Sigma _{jj} = || \mathbf{{X}}_j ||^2_2 = 1 \; \forall j$. Therefore, ensuring that the knockoff features *K* exhibits the same covariance structure as the original features *X* will be through:
  16 $$\begin{aligned} \mathbf{{K}}^T \;\mathbf{{K}} = \mathbf{{\Sigma }}, \qquad \mathbf{{X}}^T \; \mathbf{{K}} = \mathbf{{\Sigma }} - diag\{\mathbf{{s}}\} \ \end{aligned}$$
  where **s** is a *p*-dimensional non-negative vector. Moreover, the correlation between distinct original and knockoff features are the same as the correlation between the originals, because $\mathbf{{\Sigma }}$ and $\mathbf{{\Sigma }} - diag \{\mathbf{{s}} \}$ are equal on off-diagonal entries: $\mathbf{{X}}^T_j\;\mathbf{{K}}_k = \mathbf{{X}}^T_j\;\mathbf{{X}}_k \forall \; j \ne k.$ However, comparing the knockoff feature *K* with its original *X*, we observe that: $\mathbf{{X}}^T_j\;\mathbf{{K}}_j = \Sigma _{jj} - s_j = 1- s_j$, where $\mathbf{{X}}^T_j \;\mathbf{{X_j}} = \mathbf{{K}}^T_j \; \mathbf{{K}}_j = 1$. To ensure that knockoff filter does not sacrifice the power in detecting signals, the entries of $\mathbf{{s}}$ need to be as large as possible so that $\mathbf{{X}}_j$ is not too similar to its corresponding knockoff $\mathbf{{K}}_j.$
- *Statistic for each pair of original and knockoff* After generating the knockoff variables, the knockoff variables will be augmented to the original design matrix to form an $n \times 2p$ matrix $[\mathbf{{X}}\;\mathbf{{K}} ]$. Variable importance statistic $W_j$ will be calculated to help differentiate between those variables that will be included in the model and those variables that will not be included. $W_j$ is computed for each $\beta _j$, $j = 1, \ldots , p$, and large positive values of *w* is an evidence against the null hypothesis that $\beta _j = 0$. The knockoff filter method considers the Lasso [12] model, and $\ell _{1}$ norm penalized regression given by:
  17 $$\hat{\beta }(\lambda ) = \arg \min _{b} \left\{ {\frac{1}{2}||{\mathbf{y}} - {\mathbf{Xb}}||_{2}^{2} + \lambda ||{\mathbf{b}}||_{1} } \right\}$$
  On the Lasso path, taking $Z_j$ to be the point $\lambda$ at which feature $\mathbf{{X}}_j$ first enters the mode,
  18 $$\begin{aligned} Z_j = \sup \{\lambda : \hat{\beta }_j(\lambda ) \ne 0 \} \end{aligned}$$
  $Z_j$ is computed on the augmented $n \times 2p$ design matrix $[\mathbf{{X}}\;\mathbf{{K}} ]$ which yields a 2*p*-dimensional vector $(Z_1, \dots , Z_{2p} )$ and $W_j$ is obtained by:
  19 $$\begin{aligned} W_j = Z_{jx} \vee Z_{jk} {\left\{ \begin{array}{ll} +1, & Z_{jx}> Z_{jk} \\ -1, & Z_{jx} < Z_{jk} \end{array}\right. } \end{aligned}$$
  where $Z_{jx}$ is the statistic for each original feature in $\mathbf{{X}}$ and $Z_{jk}$ is the statistic for each corresponding knockoff feature in $\mathbf{{K}}$. A large positive value of $W_j$ means that the original variable $X_j$ enters the Lasso model early before its corresponding knockoff, which indicates that this variable is significant and should be included in the model. Other test statistic can be considered if they satisfy the flip-sign property indicated by Model-X knockoff [2], which ensures that swapping the variable with its knockoff changes the sign of $W_j$. This statistic will be replaced in our proposed model by a different one which will be explained in details in the next section.
- *FDR control* After obtaining $W_{j}$ by subtracting each pair of $Z_{j}$; the original and knockoff. The FDR is defined to be the expected value of the false discovery proportion. Therefore, knockoff method states: For any $q \in [0,1]$, the knockoff method satisfies[1]:
  20 $$\begin{aligned} FDR = \mathbb {E} \left[ \frac{\# \{ j: \beta _{j} = 0 \quad \text {and} \quad j \in \hat{S}\}}{\#\{j: j \in \hat{S} \} \vee 1 } \right] \le q \end{aligned}$$
  The denominator in Eq. 20 ensures that the fraction will equal to zero if no variables are selected, and $\hat{S}$ is defined as the selected model based on $W_j$:
  21 $$\begin{aligned} \hat{S} = \{ j: W_j \ge T \} \end{aligned}$$
  Where T is the data-dependent threshold. This data-dependent threshold is defined to ensure the FDR control. Choose a threshold $\tau>0$ such that:
  22 $$\begin{aligned} \tau = \min \{t>0: \frac{1+ \# \{j: W_j \le - t\}}{\#\{j: W_j \ge t \}} \le q \} \end{aligned}$$
  Where *q* is the targeted FDR level. Knockoff filter method guarantees that the selection rule controls the FDR at any desired *q* level if its FDR is guaranteed to be at most *q*, no matter the value of the coefficients $\beta$. In the hypothesis testing setting, we would be interested in the *p* hypotheses $H_j: \beta _j =0$ and wish to find a multiple comparison procedure that rejects individual hypotheses while controlling the FDR. If $H_j$ is is rejected, that means the data provide evidence against $H_j$ and feature *j* is selected to be in the model.

### Variable importance measures

As mentioned in the previous section that knockoff filter framework uses the Lasso test statistic as a variable importance measure. Since our proposed method is based on gradient boosting trees, in this section, we will introduce a statistic adapted to the tree models, namely; SHapely Additive exPlanations (SHAP) [8]. Complex models like the ensemble methods are not easy to understand their model and how it predicts the response variable *y*. SHAP is a game theoretic approach to calculate an additive feature importance score for each particular prediction with some properties (local accuracy, missingness and consistency). Let *f* in Eq. 1 be the original prediction model to be explained and the explanation model in an additive feature attribution methods is *g*(*z*) and it is defined as:

23 $$\begin{aligned} g(z) = \phi _0 + \sum _{i=1}^M \phi _i z_i \end{aligned}$$

Where $z \in \{0,1\}^M$ is a binary *M*- dimensional of input features, where $z=1$ if the variable is observed, $z=0$ if the variable is not observed, and $\phi _i$ is the feature attribution value. This feature attribution value $\phi _i$ can be obtained by:

24 $$\phi _{i} = \sum _{{S \subseteq p\backslash \{ i\} }} \frac{{|S|!\left( {p - |S| - 1} \right)!}}{{p!}}\left[ {f(S \cup \{ i\} ) - f(S)} \right]$$

where *S* is the subset of the features used in the model, *p* is the total number of features in the data set, *x* is the vector of features values of the instance to be explained, *f*(*S*) is the prediction for feature values in set *S* that are marginalized over features that are not included in set *S*, and *f*(*S*) is defined as the conditional expectation $E[f(X)|X_s]$.

SHAP provides a global interpretation using aggregation of the shapely values. The variable selection and feature importance can be obtained by computing the SHAP values for all the data set and summing the absolute values across all the data. SHAP allows us to understand which features contribute to the model outcome and the extent to which they influence. In practice, SHAP calculation for an $n \times p$ design matrix generates another $n \times p$ matrix with the marginal feature contribution for each instance in the data. Following the same framework of knockoff filter, we will replace the Lasso measure by the SHAP measure instead to allow us understand the black-box of the GBDT models. Therefore, SHAP values will be computed for each feature in the augmented $(n \times 2p)$ matrix- (original and knockoff), and it will be used as a statistic to compare between the original variables and their corresponding knockoffs.

### Variable selection via knockoffs using GBDT

Our proposed framework/algorithm is motivated by the original knockoff filter framework and it can be summarized as follows:

**Algorithm 1**Variable selection via knockoffs using GBDT framework 
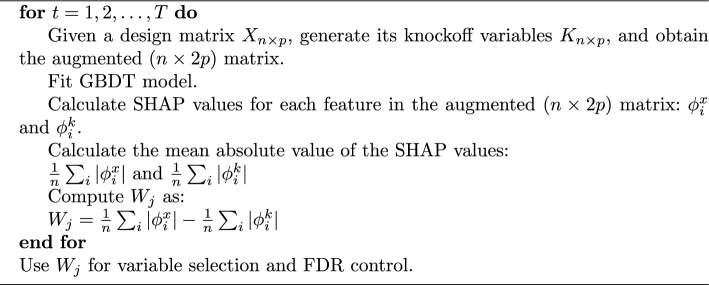


## Simulation studies

We simulated data for regression and classification settings under both linear and nonlinear associations. The design matrix *X* maintained a consistent covariance structure across all simulations, with variations in the response variable *y* and coefficients $\beta$ for regression, binary classification, and multi-class classification. We ran 100 iterations to ensure robustness. The number of variables (*p*) was fixed at 1000, while sample sizes (*n*) varied across 500, 700, 1000, 2000, and 5000, covering scenarios where $n<p$, $n=p$, and $n>p$.

For performance comparison, we applied both LightGBM and XGBoost, tuning parameters via Bayesian optimization for optimal efficiency. All analyses were conducted in R [9] using the Knockoff R package. Alongside LightGBM and XGBoost, we included the original knockoff framework for comparison. Model performance was evaluated based on false discovery rate (FDR), power, and computational time. FDR quantifies the proportion of incorrectly selected non-significant variables, estimated via false discovery proportion (FDP). Power measures the proportion of correctly identified significant variables, while computational time reflects average runtime per model in seconds.

### Linear association case

#### Regression

Consider the design matrix $X \in \mathbb {R}^{n \times p}$. Each row vector in *X* is generated as $x_i \sim \mathcal {N}_p(0, \mathbf{{\Sigma }})$ with a block dependence structure $\mathbf{{\Sigma }} = diag (\Sigma _1, \Sigma _2, \dots )$ where each $\Sigma _l$ is a $(10 \times 10)$ matrix, with the matrix elements $\sigma _{j,k} = \rho ^{|j-k|}$, where $\rho = 0.1$. The response vector was generated using the model:

25 $$\begin{aligned} \mathbf{{y}} = \mathbf{{X}} \beta + \epsilon \end{aligned}$$

Where $\beta = (2,2,2,2,2,2,2,2,2,2, 0, \dots , 0 )^T$ is the coefficients vector which indicates that only the first 10 variables are significant, and $\varvec{\epsilon }\sim \mathcal {N}_p(0,\textbf{I}_p )$. FDR was targeted at level 10$\%$. Table 1 shows the FDR estimate over 100 iterations, which is the average of FDP and standard deviation of the FDP.

**Table 1:** The FDR estimate for original knockoff, XGBoost and LightGBM for regression

	FDR		
Sample size	Original knockoff	XGBoost	LightGBM
500	0.0948 (0.1114)	0.0809 (0.0933)	0.0537 (0.0884)
700	0.0881 (0.1157)	0.0888(0.1161)	0.0537 (0.0996)
1000	0.0732 (0.0918)	0.0899 (0.1189)	0.0835 (0.1044)
2000	0.1038 (0.1180)	0.0876 (0.1054)	0.0581 (0.0787)
5000	0.0651 (0.0864)	0.0952 (0.1071)	0.0650 (0.0904)

**Table 2:** The power estimate for original knockoff, XGBoost and LightGBM for regression

	Power		
Sample size	Original knockoff	XGBoost	LightGBM
500	1	1	1
700	1	1	1
1000	1	1	1
2000	1	1	1
5000	1	1	1

The results show that LightGBM consistently outperforms XGBoost across all simulations, benefiting from GOSS and EFB to achieve a lower false discovery rate (FDR). LightGBM also handles high-dimensional data well, even when $n < p$, and outperforms the original knockoff method, particularly in controlling FDR. For instance, at $n = 500$, LightGBM achieves an FDR of 5% compared to 9.5% for knockoff with Lasso, both within the 10% target but favoring LightGBM.

**Fig. 2:**
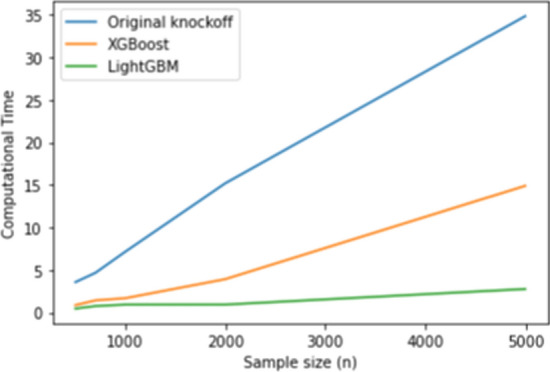
Computational time (s) for original knockoff, XGBoost and LightGBM in the regression setting

Table 2 confirms that all methods perform well, underscoring SHAP’s effectiveness in feature attribution. LightGBM’s low FDR and 100% power indicate strong null hypothesis control while maintaining high sensitivity. Additionally, Figure 2 shows that LightGBM has the lowest computational time, whereas knockoff with Lasso is the most time-intensive, especially for large $n$.

#### Binary classification

Consider the design matrix $X \in \mathbb {R}^{n \times p}$. Each row vector in *X* is generated as $x_i \sim \mathcal {N}_p(0, \mathbf{{\Sigma }})$ with a block dependence structure $\mathbf{{\Sigma }} = diag (\Sigma _1, \Sigma _2, \dots )$ where each $\Sigma _l$ is a $(10 \times 10)$ matrix, with the matrix elements $\sigma _{j,k} = \rho ^{|j-k|}$, where $\rho = 0.1$.

The response variable is generated using the logistic model:

26 $$\begin{aligned} {Pr(y_i=1|x_i)} = \frac{\exp (x_{i}^{'} \beta )}{1 + \exp (x_{i}^{'} \beta )} \end{aligned}$$

Where $\beta = (2,2,2,2,2,2,2,2,2,2, 0, \dots , 0 )^T$ is the coefficients vector which indicates that only the first 10 variables are significant. FDR was targeted at level 10$\%$.

**Table 3:** The FDR estimate for original knockoff, XGBoost and LightGBM for binary classification

	FDR		
Sample size	Original knockoff	XGBoost	LightGBM
500	0.0768 (0.0983)	0.0743 (0.0997)	0.0726 (0.0947)
700	0.0784 (0.1138)	0.0793 (0.0996)	0.0744 (0.1071)
1000	0.0920 (0.1112)	0.0941 (0.1097)	0.0650 (0.0926)
2000	0.0692 (0.0940)	0.0654 (0.0935)	0.0606 (0.0939)
5000	0.0714 (0.0888)	0.0751 (0.0824)	0.0729 (0.1011)

Table 3 presents the FDR estimates for different sample sizes in the binary classification case, where we have only two classes. The results clearly indicate that LightGBM + SHAP exhibits the lowest FDR estimate compared to the other two methods. It is worth noting that LightGBM continues to outperform XGBoost in binary classification, just as it does in regression. This can be attributed to the advantages inherent in LightGBM that are not present in XGBoost. In terms of power, as shown in Table 4, LightGBM demonstrates comparable power across different data sizes. However, in the case where $n=500$, LightGBM exhibits relatively lower power compared to the original knockoff method. This finding is not surprising since classical methods like Lasso can sometimes outperform machine learning techniques due to their specific design characteristics.

**Table 4:** The power estimate for original knockoff, XGBoost and LightGBM for binary classification

	Power		
Sample size	Original knockoff	XGBoost	LightGBM
500	1	0.928	0.969
700	1	1	1
1000	1	1	1
2000	1	1	1
5000	1	1	1

**Fig. 3:**
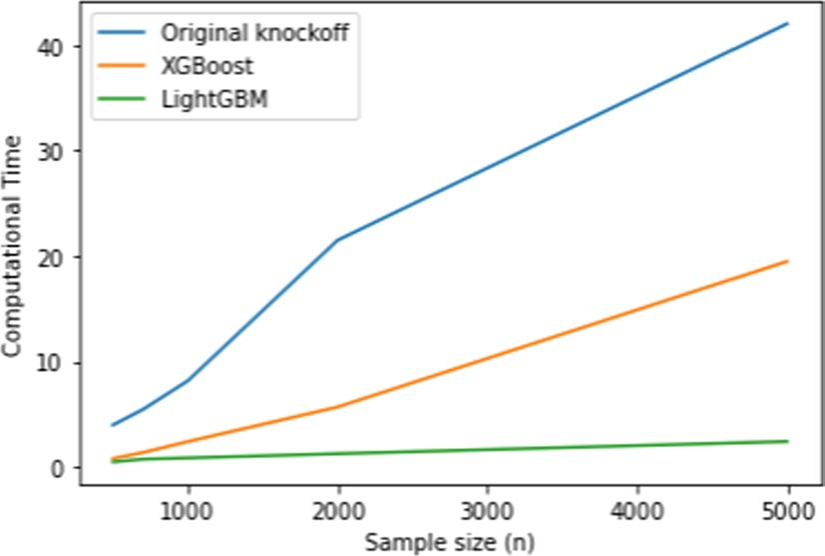
Computational time (s) for original knockoff, XGBoost and LightGBM in the binary classification

Figure 3 illustrates the relationship between sample size and computational time for the original knockoff method, XGBoost, and LightGBM. It is evident that as the sample size increases, both the original knockoff method and XGBoost experience a substantial increase in computational time. In contrast, LightGBM demonstrates the ability to maintain a low computational time even for large datasets, even when $n=5000$. This characteristic is one of the main advantages of LightGBM, as it can effectively handle large datasets while utilizing reduced memory and time, all while maintaining comparable computational power to the other methods.

#### Multi-class classification

Here, we considered the same design matrix $X \in \mathbb {R}^{n \times p}$ as in binary classification.

Let the probability that *i*-th response falls in the *j*-th class be:

27 $$\begin{aligned} \pi _{ij} = P(Y_i =j) \end{aligned}$$

The probabilities of the response were modeled by the multinomial logit model.

28 $$\begin{aligned} \pi _{ij} = \frac{\exp (\mathbf{{X}} \beta )}{\sum \exp (\mathbf{{X}} \beta )} \end{aligned}$$

Where

$$\beta = \left[ {\begin{array}{ccccccccccccccccccccccc} 3 & 3 & 3 & 3 & 3 & 3 & 3 & 3 & 3 & 3 & 0 & 0 & 0 & 0& 0 & 0 & 0 & 0 & 0 & 0 & 0 & \dots & 0\\ 0 & 0 & 0 & 0 & 0 & 0 & 0 & 0 & 0 & 0 & 2 & 2 & 2 & 2 & 2 & 2 & 2 & 2 & 2 & 2 & 0 & \dots & 0 \\ \end{array} } \right] ^T$$

The data were simulated in the multi-class classification case (3-classes) such that the first class is explained by the first 10 features and the second class is explained by the second 10 features. In other words, the beta coefficients is designed such that the first 10 features (1 : 10) are significant for the first class while the second 10 features (11 : 20) are relevant to the second class only. The probabilities for the first two classes were estimated using the multinomial distribution, and the third class probabilities were calculated using the fact that the sum of probabilities must add up to 1. FDR was targeted at level 10$\%$.

**Table 5:** The FDR estimate for original knockoff, XGBoost and LightGBM for multi-class classification (3 classes)

	FDR		
Sample size	Original knockoff	XGBoost	LightGBM
500	0.0809 (0.0823)	0.0796 (0.0715)	0.0683 (0.0712)
700	0.0798 (0.0823)	0.0938 (0.0857)	0.0756 (0.0767)
1000	0.1063 (0.0819)	0.0873 (0.0907)	0.0688 (0.0678)
2000	0.0880 (0.0772)	0.0931 (0.0798)	0.0755 (0.0777)
5000	0.0760 (0.0828)	0.0860 (0.0740)	0.0738 (0.0719)

Table 5 presents the results of the multi-class classification setting, where we have three classes. The table demonstrates that LightGBM is capable of effectively modeling datasets of various sizes, outperforming both XGBoost and the original knockoff method across all scenarios, including cases where $n<p$, $n>p$, and $n=p$. LightGBM has demonstrated its ability to handle high-dimensional cases where $n<p$, which is representative of real-world datasets. The proposed framework allows for the control of false discovery rate (FDR) while maintaining a high power. Power, in this context, refers to the sensitivity of the model in identifying important variables within the dataset.

**Table 6:** The power estimate for original knockoff, XGBoost and LightGBM for multi-class classification (3-classes)

	Power		
Sample size	Original knockoff	XGBoost	LightGBM
500	0.9995	0.914	0.9535
700	1	0.9935	1
1000	1	1	1
2000	1	1	1
5000	1	1	1

**Fig. 4:**
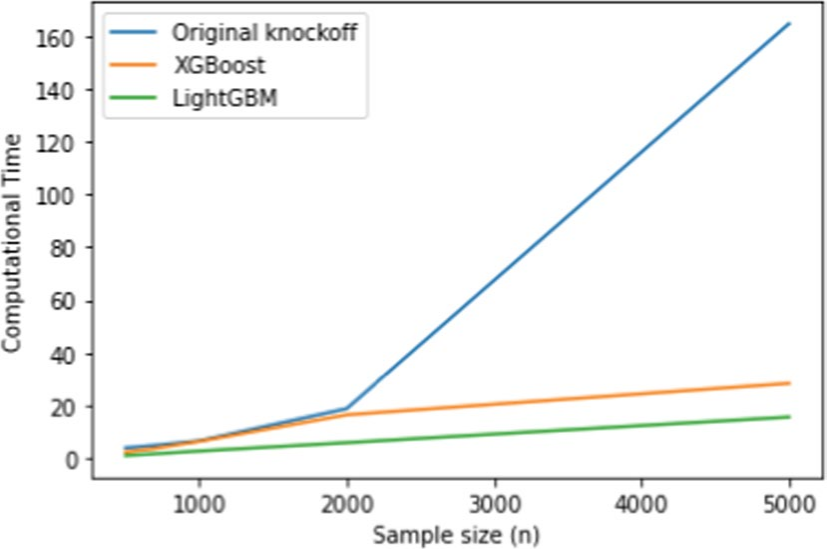
Computational time (s) for original knockoff, XGBoost and LightGBM for the multi-class classification

Table 6 shows that the proposed method using LightGBM outperforms XGBoost while have a comparable power with the Original knockoff filter method. LightGBM still has the lowest computational time even for the multi-class classification case, while the orignal kncokoff method has the highest computational time. Figure 4 shows the computational time in seconds for each model in training the dataset.

### Non-linear association case

#### Regression

Consider the design matrix $X \in \mathbb {R}^{n \times p}$. Each row vector in *X* is generated as $x_i \sim \mathcal {N}_p(0, \mathbf{{\Sigma }})$ with a block dependence structure $\mathbf{{\Sigma }} = diag (\Sigma _1, \Sigma _2, \dots )$ where each $\Sigma _l$ is a $(10 \times 10)$ matrix, with the matrix elements $\sigma _{j,k} = \rho ^{|j-k|}$, where $\rho = 0.1$. The response variable was generated similar to the model in Eq. 25 but replacing $\mathbf{{X}}$ by $\mathbf{{X}}^{2}$. FDR was targeted at level 10$\%$.

We omitted the original knockoff filter from the comparison because Lasso was designed for linear scenarios, and its effectiveness diminishes considerably when applied to non-linear situations. It tends not to report significance for the higher order terms. Table 7 shows the FDR estimate for both XGBoost and LightGBM under the nnonlinear association case. LightGBM is performing very well in high-dimensional cases in terms of controlling the FDR under the desirable level $10\%$. LightGBM has the lowest FDR than XGBoost for all cases of n.

**Table 7:** The FDR estimate for XGBoost and LightGBM for regression (nonlinear)

	FDR	
Sample size	XGBoost	LightGBM
500	0.0657 (0.1035)	0.0622 (0.0795)
700	0.0752 (0.0973)	0.0693 (0.0893)
1000	0.0963 (0.1174)	0.0771 (0.1115)
2000	0.0810 (0.1054)	0.0816 (0.1122)
5000	0.0739 (0.0939)	0.0613 (0.0885)

**Fig. 5:**
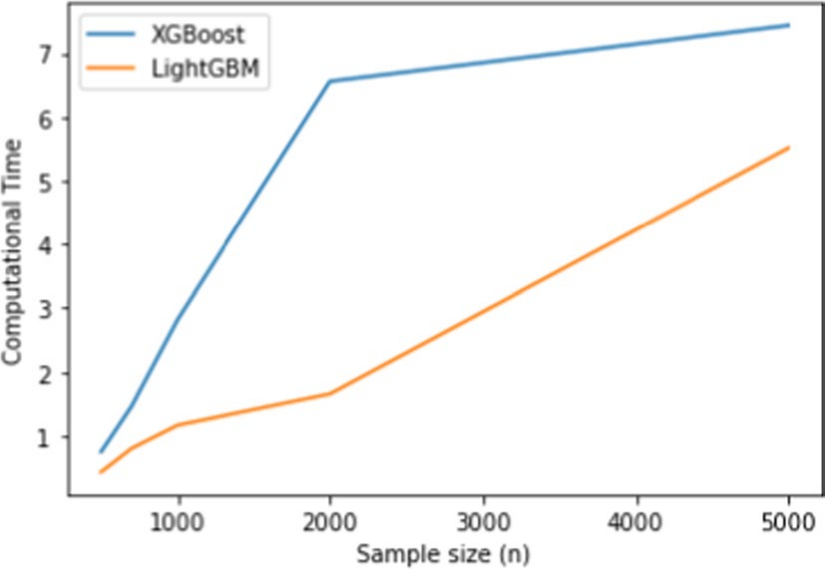
Computational time (s) for XGBoost and LightGBM for the regression setting (nonlinear)

Table 8 shows that our proposed method outperforms XGBoost for how sensitive it is to detect the significant variables. The power for XGBoost when $n=500$ is about $62 \%$ and it increases to $99 \%$ when $n=700$ while the power for LightGBM is $100 \%$ for all cases. That indicates that LightGBM is more powerful for identifying the important variables related to the model. Figure 5 shows that LightGBM requires less computational time than XGboost, and it is much faster than XGboost especially for big data.

**Table 8:** The power estimate for XGBoost and LightGBM for regression (nonlinear)

	Power	
Sample size	XGBoost	LightGBM
500	0.619	1
700	0.99	1
1000	1	1
2000	1	1
5000	1	1

#### Binary classification

Consider the design matrix $X \in \mathbb {R}^{n \times p}$. Each row vector in *X* is generated as $x_i \sim \mathcal {N}_p(0, \mathbf{{\Sigma }})$ with a block dependence structure $\mathbf{{\Sigma }} = diag (\Sigma _1, \Sigma _2, \dots )$ where each $\Sigma _l$ is a $(10 \times 10)$ matrix, with the matrix elements $\sigma _{j,k} = \rho ^{|j-k|}$, where $\rho = 0.1$.

The response variable is generated using the following function:

29 $$\begin{aligned} f(x) = 2X_1^2 + 2X_2^2 - 2X_3^2 - 2X_4^2 + 2X_5- 2X_6 + 2X_7- 2X_8 +2X_9 + 2X_{10} \end{aligned}$$

For each observation *x*, the class label is obtained based on the probability from the Bernoulli distribution, with $p(x) \propto f(x)$. The classification is defined using only the first ten variables.

**Table 9:** The FDR estimate for XGBoost and LightGBM for binary classification (nonlineaar)

	FDR	
Sample size	XGBoost	LightGBM
500	0.1475 (0.1676)	0.1094 (0.1537)
700	0.0733 (0.1241)	0.0724 (0.1120)
1000	0.0810 (0.0965)	0.0784 (0.0935)
2000	0.0783 (0.1001)	0.0773 (0.1007)
5000	0.0820 (0.0912)	0.0736 (0.0909)

Table 9 shows that LightGBM consistently achieves the lowest FDR in binary classification with nonlinear data, staying below the 10% target, while XGBoost reaches 15% at $n=500$. Table 10 further confirms LightGBM’s superior power, especially when $n \le p$. As expected, both methods perform well with larger $n$, but LightGBM excels in identifying important variables.

**Table 10:** The power estimate for XGBoost and LightGBM for binary classification (nonlinear)

	Power	
Sample size	XGBoost	LightGBM
500	0.652	0.866
700	0.576	0.95
1000	0.989	1
2000	1	1
5000	1	1

**Fig. 6:**
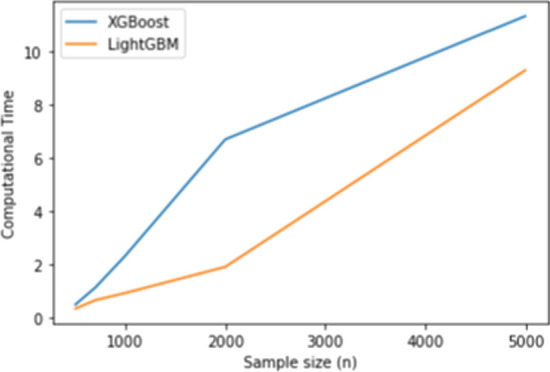
Computational time (s) for XGBoost and LightGBM for the binary classification (nonlinear)

Figure 6 shows that LightGBM is much faster than XGBoost for the binary classification - nonlinear case.

#### Multi-class classification

Here, we considered the same design matrix $X \in \mathbb {R}^{n \times p}$ as in binary classification.

A data set is simulated for the three-class example for the nonlinear case by defining the following two functions:

30 $$\begin{aligned} f_1(x)&= 2 X_1 + 2 X_3^2 - 0.2X_5 + 0.4 X_7 + 3X_9 \end{aligned}$$

31 $$\begin{aligned} f_2(x)&= X_1^2 + 0.6X_2 - 0.5X_4^2 + 2X_6 + 2.5X_8 - 3X_{10} \end{aligned}$$

For each observation *x*, the class label is given using the multinomial sampling of ($p_1(x)$, $p_2(x)$) with $p_k(x) \propto f_k(x)$, $k=1,2$. The classification is defined by $x_1$, $x_3^2$, $x_5$, $x_7$ and $x_9$ for the first class. The second class is defined by $x_1^2$, $x_2$, $x_4^2$, $x_6$, $x_8$ and $x_{10}$. The third class probability is computed by the fact that the sum of the probabilities must add up to 1. The FDR targeted level is $20\%$.

Table 11 shows the FDR estimate for the multi-class classification (nonlinear case). This simulation was a bit complex, so we allowed the FDR to be $20\%$. XGBoost and LightGBM were able to have the FDR estimate under control, under $20 \%$. LightGBM is performing noticeably better than XGBoost in terms of having the lowest FDR. The highest FDR using XGBoost was $19 \%$, while the highest using LightGBM was $15 \%$.

**Table 11:** The FDR estimate for XGBoost and LightGBM for multi-class classification (nonlineaar)

	FDR	
Sample size	XGBoost	LightGBM
500	0.1991 (0.1715)	0.1564 (0.1329)
700	0.1516 (0.1232)	0.1486 (0.1282)
1000	0.1526 (0.1252)	0.1522 (0.1201)
2000	0.1735 (0.1258)	0.1439 (0.1257)
5000	0.1729 (0.1300)	0.1552 (0.1324)

As shown in Table 12, LightGBM has the highest power in all cases. XGBoost performed poorly in the nonlinear multi-class classification case. The highest power that XGBoost achieved even with large *n* was $50\%$, that meas XGBoost was only able to detect on average 5 variables out of 10 to be significant.

**Table 12:** The power estimate for XGBoost and LightGBM for multi-class classification (nonlinear)

	Power	
Sample size	XGBoost	LightGBM
500	0.421	0.902
700	0.485	0.978
1000	0.5	0.997
2000	0.5	1
5000	0.5	1

**Fig. 7:**
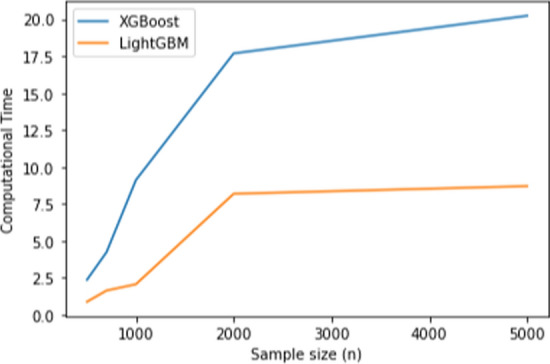
Computational time (s) for XGBoost and LightGBM for the multi-class classification (nonlinear)

LightGBM uses the exclusive feature bundling which reduces dimensionality and saves a lot of memory and time. As shown in Fig. 7 LightGBM has the lowest computational time than XGBoost.

## Microarray data analysis

We extended our framework to analyze the prostate cancer dataset, which was obtained from [11]. The dataset can be accessed in R through the “sda" package, specifically in a data file named “singh2002". It comprises a total of 102 cases and includes 6033 genes. Among the cases, 52 correspond to prostate cancer patients, while the remaining cases are classified as normal patients. The expression levels of the same 6033 genes were measured for each case. Our analysis aims to detect the most significant genes that contribute in classifying patients with prostate cancer. FDR was targeted at $10\%$. The index numbers of the genes selected are summarized in Table 13.

**Table 13:** Selected genes on prostate cancer data

Selected genes via knockoffs using LightGBM	
2, 37, 60, 63, 77, 221, 225, 285, 406, 411, 519, 571, 610, 653, 721, 788, 888, 897, 913, 979, 1022, 1053, 1057, 1061, 1134, 1147, 1322, 1392, 1469, 1492, 1511, 1515, 1550, 1627, 1631, 1720, 1957, 2020, 2191, 2203, 2327, 2605, 4012, 4405, 5000, 5170, 5530, 5568, 5686, 6027	

Our analysis revealed a larger number of significant genes compared to those reported in the literature [3]. However, we did identify some common genes that overlapped with the literature findings. To further validate our results, we conducted an additional step. We extracted the significant variables we identified in our analysis, as well as the genes identified in the literature. Using random forest, we assessed the ability of both sets of genes to explain the variability in the data and classify the patients. Surprisingly, our reduced model consisting of only 50 genes achieved a $100\%$ correct classification rate. To validate the comparison between the group of genes we discovered and the number of genes found in the literature, we utilized four measures.

**Table 14:** Assessment measures using both models

	Using the proposed framework	Found in the literature
Accuracy	$100\%$	$93\%$
Sensitivity	$100\%$	$86\%$
Specificity	$100\%$	$100\%$
Kappa	1	0.8667

Accuracy was used to assess the classifier’s performance in identifying prostate cancer patients. Table 14 shows that our method achieved 100% accuracy, outperforming the 93% accuracy of genes selected from the literature. Sensitivity (true positive rate) was also 100% with our method, compared to 86% for literature-based genes. Specificity (true negative rate) was perfect for both approaches. The kappa statistic, measuring classification agreement beyond chance, was 1 for our method, indicating strong reliability, while the literature-based model had a lower score of 0.87. These results highlight the superiority of our approach in selecting genes for accurate prostate cancer classification.

## Conclusion

Variable selection is a fundamental challenge in statistics, especially in high-dimensional settings where handling a large number of variables is impractical. The goal is to retain only those variables that meaningfully contribute to model performance while maintaining a low false discovery rate. The knockoff filter provides an effective way to achieve this by generating synthetic control variables that help distinguish between significant and irrelevant features. In this study, we integrated knockoffs with LightGBM, leveraging SHAP values to enhance interpretability. Our results demonstrate that this approach effectively selects important variables while maintaining statistical rigor and computational efficiency.

While our method performs well across different simulation scenarios and real-world datasets, there are areas for future improvement. Investigating interactions between covariates and refining feature selection at the class level in multi-class problems could further enhance accuracy. Additionally, extending this framework to other machine learning architectures could provide even more robust solutions for variable selection in complex datasets.

## Data Availability

The Singh et al. (2002) prostate cancer dataset used in this study is publicly available and can be accessed through the *sda* R package (rdrr.io) or the *DBCRepository* (leo.ugr.es). Researchers can obtain the dataset following the respective repository guidelines.

## Appendix

### Identifying the important variables for each class in the multi-class classification

SHAP helps quantify how much each variable contributes to a model’s predictions for every instance in an $n \times p$ dataset, resulting in an $n \times p$ SHAP matrix. In multi-class classification, this extends to producing a separate $n \times p$ matrix for each class, allowing us to see which variables matter not just overall but for each specific class. We estimate the false discovery rate (FDR) and power in detecting important variables in a three-class setting under both linear and nonlinear models. Traditional methods don’t offer this level of insight–SHAP makes it possible by explaining machine learning predictions.

#### Linear case

Let the probability that *i*-th response falls in the *j*-th class be:

32 $$\begin{aligned} \pi _{ij} = P(Y_i =j) \end{aligned}$$

The probabilities of the response were modeled by the multinomial logit model.

33 $$\begin{aligned} \pi _{ij} = \frac{\exp (\mathbf{{X}} \beta )}{\sum _{k=1}^{J} \exp (\mathbf{{X}} \beta )} \end{aligned}$$

Where

$$\beta = \left[ {\begin{array}{ccccccccccccccccccccccc} 3 & 3 & 3 & 3 & 3 & 3 & 3 & 3 & 3 & 3 & 0 & 0 & 0 & 0& 0 & 0 & 0 & 0 & 0 & 0 & 0 & \dots & 0\\ 0 & 0 & 0 & 0 & 0 & 0 & 0 & 0 & 0 & 0 & 2 & 2 & 2 & 2 & 2 & 2 & 2 & 2 & 2 & 2 & 0 & \dots & 0 \\ \end{array} } \right] ^T$$

The data for the 3-class classification were generated so that the first 10 features ($X_1$–$X_{10}$) define the first class, while the next 10 ($X_{11}$–$X_{20}$) define the second. Probabilities for the first two classes were assigned via a multinomial distribution, with the third ensuring the sum equals 1. The target FDR was 10%.

Table 15 presents the FDR and power estimates for the linear case using LightGBM with SHAP. Power is near 100% when $n \ge p$. In high-dimensional settings ($n=500$), power drops to 84% for the first class and 51% for the second but improves to 99% and 87%, respectively, when $n=700$.

**Table 15:** The FDR and power estimate per each class for multi-class classification using LightGBM (linear)

	First class		Second class	
Sample size	FDR	Power	FDR	Power
500	0.0836	0.845	0.1038	0.512
700	0.0773	0.99	0.1108	0.87
1000	0.0703	1	0.1118	0.98
2000	0.0812	1	0.2716	1
5000	0.1257	1	0.2106	1

#### Nonlinear case

For the three-class nonlinear case, data is simulated using the functions:

34 $$\begin{aligned} f_1(x)&= 2 X_1 + 2 X_3^2 - 0.2X_5 + 0.4 X_7 + 3X_9 \end{aligned}$$

35 $$\begin{aligned} f_2(x)&= X_1^2 + 0.6X_2 - 0.5X_4^2 + 2X_6 + 2.5X_8 - 3X_{10} \end{aligned}$$

Class labels are assigned via multinomial sampling with probabilities $p_k(x) \propto f_k(x)$ for $k=1,2$, and $p_3(x)$ determined to sum to 1. The first class depends on $X_1, X_3^2, X_5, X_7, X_9$, while the second depends on $X_1^2, X_2, X_4^2, X_6, X_8, X_{10}$. The target FDR is 20%.

Table 16 reports the power and FDR estimates for multi-class classification using LightGBM with SHAP. The power for identifying key variables is 100% when $n \ge p$, dropping to 92% when $n=700$. Even in high-dimensional settings, the power remains strong, indicating the significance of identified variables.

**Table 16:** The FDR and power estimate per each class for multi-class classification using LightGBM (nonlinear)

	First class		Second class	
Sample size	FDR	Power	FDR	Power
500	0.2193	0.93	0.1964	0.7877
700	0.2027	0.99	0.1490	0.92
1000	0.2412	1	0.1731	0.99
2000	0.2398	1	0.2191	1
5000	0.2605	1	0.2226	1
